# Carboxymethyl Cellulose as a Food Emulsifier: Are Its Days Numbered?

**DOI:** 10.3390/polym15102408

**Published:** 2023-05-22

**Authors:** Eduardo M. Costa, Sara Silva, Carla F. Pereira, Alessandra B. Ribeiro, Francisca Casanova, Ricardo Freixo, Manuela Pintado, Óscar L. Ramos

**Affiliations:** CBQF—Centro de Biotecnologia e Química Fina—Laboratório Associado, Escola Superior de Biotecnologia, Universidade Católica Portuguesa, Rua Diogo Botelho 1327, 4169-005 Porto, Portugal

**Keywords:** carboxymethyl cellulose, gut inflammation, co-culture inflammation model, IL-6, IL-8, TNF-α

## Abstract

Carboxymethyl cellulose use in industry is ubiquitous. Though it is recognized as safe by the EFSA and FDA, newer works have raised concerns related to its safety, as in vivo studies showed evidence of gut dysbiosis associated with CMC’s presence. Herein lies the question, is CMC a gut pro-inflammatory compound? As no work addressed this question, we sought to understand whether CMC was pro-inflammatory through the immunomodulation of GI tract epithelial cells. The results showed that while CMC was not cytotoxic up to 25 mg/mL towards Caco-2, HT29-MTX and Hep G2 cells, it had an overall pro-inflammatory behavior. In a Caco-2 monolayer, CMC by itself increased IL-6, IL-8 and TNF-α secretion, with the latter increasing by 1924%, and with these increases being 9.7 times superior to the one obtained for the IL-1β pro-inflammation control. In co-culture models, an increase in secretion in the apical side, particularly for IL-6 (692% increase), was observed, and when RAW 264.7 was added, data showed a more complex scenario as stimulation of pro-inflammatory (IL-6, MCP-1 and TNF-α) and anti-inflammatory (IL-10 and IFN-β) cytokines in the basal side was observed. Considering these results, CMC may exert a pro-inflammatory effect in the intestinal lumen, and despite more studies being required, the incorporation of CMC in foodstuffs must be carefully considered in the future to minimize potential GI tract dysbiosis.

## 1. Introduction

Carboxymethyl cellulose (CMC) is a water-soluble polysaccharide obtained from cellulose through a Williamson etherification, which has long been used in food, cosmetics, and pharmaceutical industries [1,2]. Structurally, CMC is a copolymer constituted by β-ᴅ-glucose and β-ᴅ-glucopyranose 2-*O*-(carboxymethyl)-monosodium salt units linked by β-1,4-glycosidic bonds, in which the insertion in the backbone of carboxymethyl groups (-CH_2_-COOH) on the glucose residue improves its interfacial characteristics [3,4]. With this modification, CMC acquires new physicochemical and functional properties that allow its use as a thickener, stabilizer, rheology modifier, gelling, water retention agent and emulsifier in food products and, namely, its usage as a dietary emulsifier.

Dietary emulsifiers are, by definition, compounds that enable the homogenization of immiscible compounds, which are commonly present in foodstuffs as they are used to improve texture and extend shelf life [5]. They are defined in the Codex Alimentarius as additives that form or maintain a uniform emulsion of two or more phases in a food, possess hydrophilic and hydrophobic moieties and lead to reductions in the interfacial tension between oil and water phases in foodstuffs [6]. They are broadly characterized in three classes (low molecular weight emulsifiers, amphiphilic biopolymers and solid/colloidal particles), with each one encompassing different molecules and overall characteristics. Carboxymethyl cellulose, being a polysaccharide, falls into the amphiphilic biopolymers class, which is usually used as a thickening agent, thus increasing emulsion viscosity and foodstuff stability [7]. CMC’s application as a dietary emulsifier in foodstuffs is usually linked with improved food quality, good mouthfeel and increased food safety, with recent advances showing that it can be used to create nano-emulsions capable of controlling the particle size, concentration and texture of formulations and even as a delivery agent of probiotics in the colon [2]. However, in recent years, a troubling trend relating dietary emulsifiers to gut inflammation has been emerging, with CMC being at the forefront.

Within the gastrointestinal tract (GIT) resides a complex harmony between colonizing microorganisms and host cells. This is, normally, a beneficial relationship between colonizer and colonized, where the former is critical for the wellbeing of the latter, as gut microbiota meditate metabolism, drive the host immune system and impede pathogen adhesion [5,8]. When this relation is disrupted, a dysbiotic state occurs, leading to gut inflammation and diseases, such as inflammatory bowel disease (IBD), cancer, obesity and diabetes [8,9]. Herein lies CMC’s problem: dietary emulsifiers, and CMC, in particular, have been associated with gut dysbiosis in recent years. This dysbiotic activity has been presently linked to an interaction of CMC with the intestinal mucus, which causes the removal/reduction of this layer that protects intestinal cells. This, in turn, leads to bacterial encroachment in the epithelial cell layer, alterations in the gut microbiota composition and the fecal metabolome (particularly in reductions of short-chain fatty acids) and an increase of lipopolysaccharide (LPS)-producing bacteria which, all combined, result in the consequent development of chronic inflammation and associated diseases [5,8,9,10,11]. However, is this the only cause of CMC’s deleterious effects on the gut? To this question, there are presently no answers, as CMC’s classification of being Generally Recognized as Safe (GRAS) by the FDA and as a food additive for technological purposes in unrestricted dosages (E466) by the EFSA [9] led to the inexistence of any kind of studies in this topic. Thus, when one considers the ubiquitous nature of CMC in food products and the increase in correlation between CMC and gut inflammatory diseases, the lack of in vitro mechanistic data on this topic becomes worrying. Therefore, we sought to understand if CMC’s effect on gut health was only microbiota-related or if there was a direct cause and effect related to CMC’s interaction with the GI tract epithelial cells. To that end, the effect of a commercially available CMC upon the GI tract was evaluated, in vitro, in two steps: first, in monolayer models, sample capacity to impact the metabolism of GI tract cell lines and to modulate the inflammatory response of colonic cells was evaluated; second, in complex co-culture models, it was evaluated if CMC’s presence in the intestinal lumen would impact three parameters—GI tract membrane integrity, liver cell metabolism (thus simulating any deleterious systemic effect) and if it would modulate inflammatory responses in the intestinal lumen, leading to the production of metabolites in the basal side of the membrane capable of causing an inflammatory response in macrophages. With this data, we hope to gain a better understanding of how CMC impacts gut health and help drive an evidence-based discussion regarding its future use in foodstuffs.

## 2. Materials and Methods

### 2.1. Source and Preparation of CMC Solution

Carboxymethyl cellulose ([C6H7O(OH)_3−x_(OCH2COOH)_x_]_n_, substitution degree of 0.71, purity 99.5% and ***M_w_*** of 250 kDa) was obtained from Sigma (St. Louis, MI, USA). This data was experimentally determined and confirmed by Costa, Pereira [4]. Stock solutions were prepared at 50 mg/mL in deionized water through the addition of CMC powder until the desired concentration was reached. Afterwards, the solution was left stirring at room temperature until complete dissolution. The pH was adjusted to 6.5–7.5 using 0.1 M NaOH or 0.1 M HCl, as appropriate.

### 2.2. Cell Lines and Culture Conditions

Four different cell lines were assayed throughout this work. Caco-2 cells (ATCC HTB-37), Hep G2 (ATCC HB-8065) and RAW 264.7 (ATCCC TIB-71) were obtained from the American Type Culture Collection (ATCC, Manassas, VA, USA). HT29-MTX cells (ECACC 15121711) were obtained from the European Collection of Authenticated Cell Cultures. All cells were cultured at 37 °C in a humidified atmosphere of 95% air and 5% CO_2_ as monolayers. Caco-2 and HT29-MTX cells were grown in Dulbecco’s Modified Eagle’s Medium (DMEM) (ThermoScientific, Waltham, MA, USA) with 4.5 g/L glucose, L-glutamine without pyruvate (ThermoScientific, Waltham, MA, USA), containing 10% fetal bovine serum (FBS) (ThermoScientific, Waltham, MA, USA), 1% (*v*/*v*) Non-Essential Amino Acids (NEAA) (ThermoScientific, Waltham, MA, USA) and 1% (*v*/*v*) Penicillin/Streptomycin/Fungizone (ThermoScientific, Waltham, MA, USA). Hep G2 and RAW cells were grown in DMEM with 4.5 g/L glucose, L-glutamine without pyruvate containing 10% FBS and 1% (*v*/*v*) Penicillin/Streptomycin/Fungizone. Caco-2 cells were used between passages 23 and 26, HT29-MTX between passages 45 and 50, Hep G2 cells between passages 81 and 84 and RAW cells between passages 7 and 9.

### 2.3. Cytotoxicity Evaluation

Cytotoxicity evaluation was performed according to the ISO 10993-5:2009 standard, as previously described by Costa, Pereira [4] for Caco-2, HT29-MTX and Hep G2 cells. Cells were grown to 80–90% confluence, detached using TrypLE Express (ThermoScientific, Waltham, MA, USA) and seeded at 1 × 10^4^ cells/well in a 96-well microplate (Nunclon Delta, ThermoScientific, Waltham, MA, USA). After 24 h, the culture media was carefully removed and replaced with culture media supplemented with CMC at concentrations between 1.56 and 25 mg/mL. DMSO (Sigma, St. Louis, MI, USA) at 10% (*v*/*v*) in culture media was used as a death control, and plain culture media was used as growth control. After 24 h of incubation, Presto Blue (ThermoFisher, Waltham, MA, USA) was added to each well and incubated. After this period, fluorescence (Ex: 560 nm; Em: 590 nm) was measured using a microplate reader (Synergy H1, Biotek Instruments, Winooski, VT, USA). All assays were performed in quadruplicate.

### 2.4. Caco-2 Monolayer Immunomodulation

The monolayer immunomodulatory assays in Caco-2 were performed as previously described by Machado, Costa [12]. Briefly, Caco-2 cells were seeded at 2.5 × 10^5^ cells/well in a 24-well microplate and incubated for 24 h at 37 °C. Following this, the culture media was carefully replaced with media supplemented with CMC at 25 mg/mL, and the plate was re-incubated for another 24 h. As an inflammation control, IL-1β (Invitrogen, Waltham, MA, USA) was used, while, for basal activity control, plain media was used. At the end of the assay, supernatants were collected, centrifuged to remove debris, and stored at −80 °C for further analysis.

Cytokines 6 (IL-6) and 8 (IL-8) and Tumor Necrosis Factor alpha (TNF-α) detection was performed with enzyme-linked immunosorbent assay (ELISA) using the Human IL-6 Elisa Kit High Sensitivity (Abcam, Cambridge, UK), the Legend Max Human Elisa Kit IL-8 and the Legend Max Human Elisa Kit TNF-α (BioLegend, San Diego, CA, USA) according to the manufacturers’ instructions. Cytokine values were obtained in pg/mL of sample. To diminish the variability associated with any kind of proteomic-based assay, results were expressed in Relative Percentage of Production relative to the cytokine levels in the basal (non-stimulated) control. Cytokine content of the basal control was set to 100%. All assays were performed in quadruplicate.

### 2.5. Co-Culture Models

Co-culture models were performed through adaptation of the methods previously described by Antunes, Andrade [13]. Briefly, Caco-2/HT29-MTX co-cultures were seeded on the apical chamber of a 12-well Transwell (Corning, New York, NY, USA) plate in a proportion of 90:10, respectively, to reach a monolayer with a final density of 1 × 10^5^ cells/cm^2^ in each insert, and they were maintained for 21 days until assaying.

#### 2.5.1. Cell Monolayer Integrity

In all assays, membrane integrity of the different models was evaluated through transepithelial electrical resistance (TEER) using a Millicell ERS-2 Voltohmmeter (Merck, Darmstadt, Germany). It should be noted that only the monolayers with TEER values between 150 and 250 Ω·cm^2^ were selected for permeability experiments.

#### 2.5.2. Hep G2 Cells Systemic Cytotoxicity Model

The systemic cytotoxicity model was performed as previously described by Sadeghi Ekbatan, Iskandar [14] and Lammi, Zanoni [15] with some changes. Briefly, Hep G2 cells were seeded at 1.2 × 10^5^ cells/well in a 12-well plate and allowed to incubate overnight. After this period, 21-day-old Caco-2/HT29-MTX co-culture inserts were transferred from the Transwell plate and placed over the cell’s monolayer. Following this, media in the apical chamber were replaced with blank media (basal control), media with CMC at 25 mg/mL or DMSO at 10% (*v*/*v*) (stress control), and then the plate was re-incubated for 24 h. After this, Presto Blue was added to each well, the plate was re-incubated for 1 h, and then fluorescence was measured using a microplate reader. All assays were performed in quadruplicate.

#### 2.5.3. RAW Inflammatory Model

The co-culture intestinal inflammatory model was performed through adaptation of the protocol previously described by Tanoue, Nishitani [16]. Briefly, RAW cells were seeded at 8.5 × 10^5^ cells/well in a 12-well plate and allowed to adhere overnight, following which 21-day old Caco-2/HT29-MTX co-culture inserts were transferred and placed over the cell’s monolayer. Media in the apical and basal compartments were then replaced with RPMI 1640, with conditions assayed in the apical compartment being blank RPMI (basal control), RPMI with LPS (inflammation control), RPMI with CMC at 25 mg/mL and RPMI with CMC at 25 mg/mL and LPS. Membrane integrity of the model was assessed at 0, 1 and 3 h, with aliquots for immunomodulation assays being collected at the 3 h mark. After collection of the aliquots, viability of the cells present in the basal compartment was evaluated using the Presto Blue viable dye as previously described.

Cytokines production in the apical compartment (Caco-2/HT29-MTX membrane) was performed via detection of IL-6, IL-8 and TNF-α production through ELISA assays (ELISA MAX Deluxe Set Mouse Elisa Kits, BioLegend, San Diego, CA, USA), with results being given in pg/mL of sample. To diminish the variability associated with any kind of proteomic-based assay, results were expressed in Relative Percentage of Production relative to the cytokine levels in the basal (non-stimulated) control. Cytokine content of the basal control was set to 100%.

Immunomodulatory effects upon RAW cells present in the basolateral compartment were performed using a 13-analyte mouse multiplex panel (LEGENDplex, Biolegend, San Diego, CA, USA) according to the manufacturer’s instructions. Results were obtained using a BD Accuri CSampler Plus flow cytometer gated according to the multiplex manufacturer’s instructions, and results are given in pg/mL. All determinations were performed in quadruplicate.

### 2.6. Statistical Analysis

Statistical analysis was performed using IBM SPSS Statistics v21.0.0 (New York, NY, USA) software. As the data followed a normal distribution, one-way ANOVA coupled with Tukey’s post hoc test was used to assess the differences between the results observed, with differences being considered significant for *p*-values below 0.05.

## 3. Results

### 3.1. Cytotoxicity Evaluation

**Figure 1:**
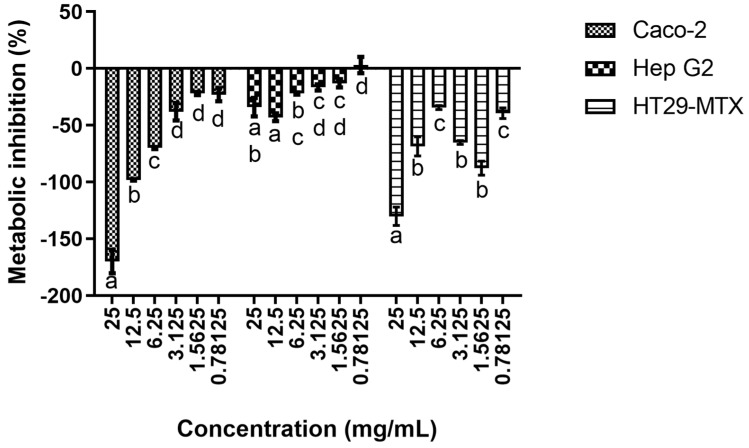
Cytotoxicity profile of CMC against the selected cell lines. Different letters represent the statistically significant differences (*p* < 0.05) found between concentrations for each cell line.

The results obtained regarding the CMC’s impact on the selected cells’ metabolic activity can be seen in Figure 1. As can be seen up to 25 mg/mL, no cytotoxic effects were observed in all tested cell lines. In fact, at higher CMC concentrations, metabolic promotions were observed, with particular emphasis being given to the high promotions registered for Caco-2 and HT29-MTX cells. When assessing the impact of the CMC concentration on the cell’s metabolism, it is possible to see that for Caco-2, a clear dose response is present for the highest concentrations tested, with a statistically significant (*p* < 0.05) effect being observed, while for the three lower concentrations tested, no clear effects were observed. For the other two cell lines, the results were more muddled, as, despite statistically significant differences (*p* < 0.05) being observed, no clear patterns of response could be ascertained.

These results are, in general, supported by the existing literature because CMC, as with most anionic polymers, has been described as being non-cytotoxic against various cell lines, mostly due to its lack of direct interaction with cells [4,17]. When considering the cell lines used in this work, the existing body of work is scarce as CMC, due to its ubiquitous use, is considered a GRAS and, thus, cytotoxicity is not normally evaluated [18]. Nevertheless, some comparisons with previous works are still possible. First, we can look into the work of Pradhan, Mulenos [19], which showed that cellulose and fibrillated cellulose materials were not cytotoxic towards Caco-2, HT29-MTX and Raji-B monolayers. When considering the effects of CMC and its derivatives upon Caco-2, CMC’s nano and microparticles have been proven to not be cytotoxic, as Javanbakht, Pooresmaeil [20] showed that hybrid CMC–copper nanoparticles were not cytotoxic. Similarly, Nwabor, Singh [21] and Javanbakht, Pooresmaeil [20] showed that alginate -CMC microcapsules had no deleterious effects towards these cell lines. For Hep G2, Cui, Si [22] showed that a CMC–casein nanocomplex was not cytotoxic; Wang, Zhang [23] showed that a CMC-based film (CMC-hydroxypropyltrimethyl ammonium chloride chitosan film) was not cytotoxic; and Wang, Zhang [24] reported that a CMC–chitosan hybrid film was not cytotoxic towards Hep G2. Similarly, El-Shafai, Ibrahim [25] showed that a CMC graphene oxide hybrid nanostructure was not cytotoxic towards Hep G2.

### 3.2. Caco-2 Monolayer Immunomodulation

**Figure 2:**
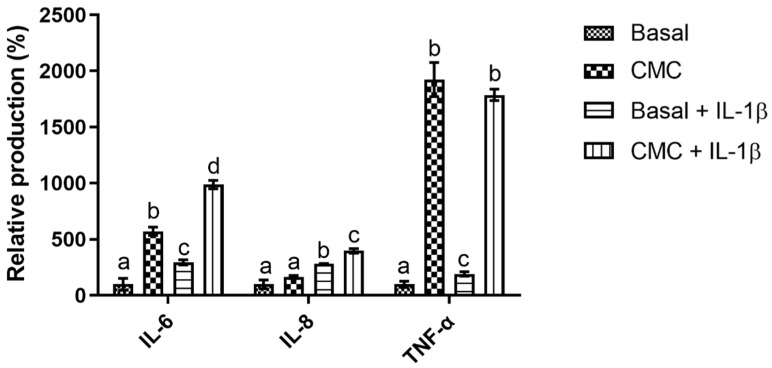
Relative percentage of cytokine production in the Caco-2 monolayer model in the tested conditions. Different letters represent the statistically significant differences (*p* < 0.05) found between conditions for each cytokine.

When evaluating the impact of CMC upon the Caco-2 monolayer system, it is possible to see (Figure 2) that it presented an overall pro-inflammatory behavior. In fact, its presence led to increases in the expression of the evaluated cytokines in all studied conditions, with an apparent synergic effect being observed in the presence of the IL1-β inflammatory stimuli.

On a cytokine per cytokine analysis, the high promotions obtained for TNF-α stand out as CMC’s presence led to statistically significant (*p* < 0.05) increases of 1924% (without inflammatory stimuli) and 1787% (with inflammatory stimuli) relative to the control. These values were 9.7 times superior to the one registered for the IL-1β stimulation, a compound that has been described as being a multifunctional cytokine that plays a major role in the initiation and amplification of inflammatory conditions through the activation of nuclear factor-kb (NF-kB) and intracellular cascades [26,27]. This behavior was also observed in the IL-6 data, where CMC’s presence also led to statistically significant (*p* < 0.05) increases of 571% and 986%, without and with IL-1β stimulation, respectively, values that were on average 2.6 times superior to those registered for the inflammation stimulation. Interestingly, and contrary to what was observed for TNF-α, statistically significant (*p* < 0.05) differences can be found for the CMC results obtained in the presence or absence of IL-1β, as the value obtained in the presence of IL-1β was significantly (*p* < 0.05) higher than the one obtained in its absence, a clear indication that a synergistic effect between CMC and the inflammation stimulation occurred. Last, but not least, for IL-8, a different pattern of response was observed, as CMC’s presence only led to significant increases in this cytokine production in the presence of IL-1β, where once again, a synergic effect was observed between the inflammatory stimuli and the tested compound but with the smaller increase being observed as it was only 1.4 times superior to that of IL-1β on its own. These results are particularly relevant as CMC has ubiquitous usage in most industries, with particular emphasis as a thickener and bulking agent in foodstuffs [4] and the promotion of cytokines associated with various intestinal diseases, such as inflammatory bowel disease (IBD), obesity and cancer [28,29], may be problematic considering its usage. Unfortunately, at present, there are no data available regarding the impact of CMC on Caco-2 and its inflammatory processes, so no comparisons can be directly drawn. Furthermore, the existing body of work regarding CMC’s impact on cells’ inflammatory processes is limited and muddled; while Nayak and Kundu [30] and Kollar, Závalová [31] reported that CMC did not lead to an increase in the secretion of pro-inflammatory cytokines in skin keratinocytes and THP-1 cells, respectively, Costa, Pereira [4] reported that four different CMC commercial samples led to strong increases in IL-6 production and small decreases in IL-8 production in skin keratinocytes. This discrepancy in results does not allow us to establish any clear reason as to how and why CMC presented the strong pro-inflammatory response in the Caco-2 monolayer model in the tested conditions. On the other hand, if one considers emulsifiers, in general, some inferences may be drawn from what has been described for carrageenan, an emulsifier that is usually considered and studied alongside CMC. For this molecule, authors have shown that it upregulates TNF-α production in Caco-2 cells through the triggering of various pathways, among which is NF-kB [32,33]. This upregulation will then induce the upregulation of IL-6 and IL-8 secretion, thus justifying the higher contents found for these cytokines [34]. This response pattern is extremely similar to the one observed here for CMC and may be the cause for the data obtained.

### 3.3. Co-Culture Models

#### 3.3.1. Systemic Cytotoxicity

**Figure 3:**
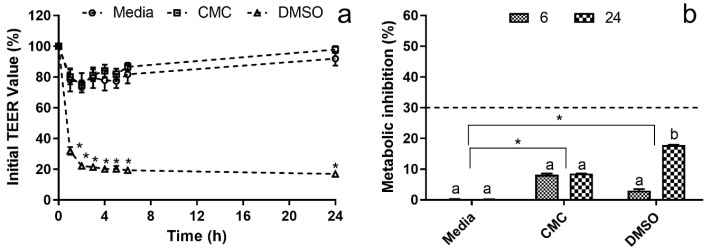
Hep G2 systemic cytotoxicity co-culture model. (**a**) Co-culture model membrane stability in the percentage of the initial TEER value. * Represents the statistically significant differences (*p* < 0.05) found between conditions in each sampling point. (**b**) Cytotoxicity profile of the evaluated samples against Hep G2 in the basolateral side of the co-culture model. The dotted line represents the 30% cytotoxicity limit as defined by the ISO 10993-5:2009 standard (ISO, 2009). Different letters represent the statistically significant differences (*p* < 0.05) found for each condition between sampling times. * Represents the statistically significant differences (*p* < 0.05) found between the test conditions and the blank (media) control.

When simulating the effects of CMC’s presence upon the intestinal lumen in barrier integrity and hepatic metabolism, the results obtained can be seen in Figure 3, and the first major takeaway is that CMC did not appear to cause any significant deleterious effects in either.

When considering CMC’s effect on intestinal barrier integrity, the data obtained (Figure 3a) showed no statistically significant (*p* > 0.05) variations in the TEER value relative to the control. In fact, CMC’s TEER values were very similar to those obtained for healthy membrane control (media only). On the other hand, for DMSO (damaged membrane), the reduction in TEER value was statistically significant (*p* < 0.05) from the onset of the assay, with an almost 70% reduction being observed within the first hour of the assay and no recovery being observed until the 24 h mark. As variations in TEER data can be directly linked with membrane health, integrity and permeability [35,36], the data obtained showed that CMC did not cause membrane rupture or irreversible permeability increases in the studied conditions. While no direct comparison of this data is possible, as no works regarding this topic were found, the results observed here are in line with the work of Pradhan, Mulenos [19], which has shown that cellulose does not cause any alterations to the barrier integrity of a Caco-2/HT29-MTX co-culture model.

When one considers the impact of the studied conditions upon the Hep G2 cells present in the basolateral compartment (Figure 3b), it is interesting to see that none of the studied conditions led to reductions in metabolic activity above the 30% threshold, defined by the ISO 10993-5:2009 standard as the cytotoxicity limit [37]. In fact, even for DMSO, which registered a steep drop in membrane integrity in the TEER data, the 30% metabolism inhibition threshold was not reached after 24 h, with an inhibition value of 17.8 ± 0.1% being observed. When analyzing the DMSO metabolism inhibition data obtained for the 6 h and the 24 h timepoints and crossing it with the TEER data, it was observed that the metabolism inhibition increased with the duration of the assay while TEER data was relatively stable from 2 h onwards. This may indicate that while the DMSO rapidly compromises membrane integrity, it does not permeate the membrane at the same rate, instead slowly permeating towards the basolateral compartment, thus leading to the increase in cytotoxicity that was observed. On the other hand, when assessing the data obtained for CMC, it can be observed that no statistically significant (*p* > 0.05) differences were found between sampling times, with a Hep G2 metabolism inhibition of ca. 10% being registered. When considering that CMC did not, apparently, alter apical membrane integrity and permeability, this data may indicate that CMC’s presence on the apical side leads to the production of some metabolite or stress that results in the viability loss registered. This is particularly evident when one considers that while CMC presented a TEER profile similar to that of the media control, it presented statistically significant (*p* < 0.05) metabolism reductions relative to the control conditions.

The results obtained for DMSO can be easily explained by the literature, as Smith, Gheux [38] showed that in co-culture models, the cytotoxicity of harmful compounds was reduced for the cells in the basolateral compartment. For CMC, the data obtained is harder to justify as no previous works addressed this topic, and the only similar work, by Pradhan, Mulenos [19], showed that the presence of cellulose, not carboxymethyl cellulose, in the apical compartment did not lead to a reduction in the viability of the Raji-B cells present in the basolateral compartment. A possible explanation for this behavior may be linked to a possible macromolecular crowding (MMC) effect, which has been described as the effect caused by the addition of polymeric materials to cell culture media, causing an excluded volume effect and leading to an increase in the deposition of cellular metabolites upon cell layer [39]. This behavior has been previously described for CMC regarding the increase of pro-collagen in skin cells [4]; and here, as cells are seeded on a permeable membrane, this may lead to the diffusion of cellular metabolites towards the basolateral compartment, thus causing the metabolism inhibition recorded.

#### 3.3.2. Immunomodulation Model

**Figure 4:**
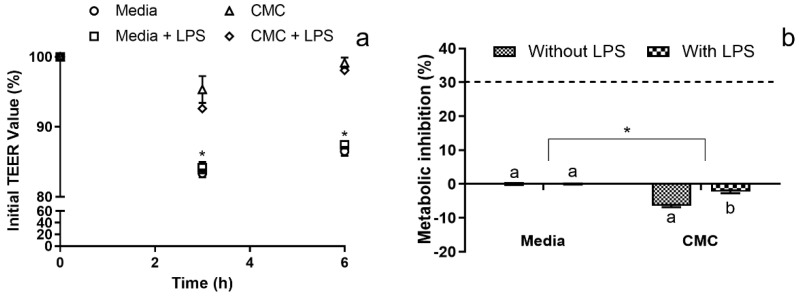
RAW co-culture model quality control parameters. (**a**) Co-culture model membrane stability in the percentage of the initial TEER value. * Represents the statistically significant differences (*p* < 0.05) found between conditions in each sampling point. (**b**) Cytotoxicity profile of the evaluated samples against the RAW in the basolateral side of the co-culture model. The dotted line represents the 30% cytotoxicity limit as defined by the ISO 10993-5:2009 standard [37]. Different letters represent the statistically significant differences (*p* < 0.05) found for each condition between sampling times. * Represents the statistically significant differences (*p* < 0.05) found between the test conditions and the blank (media) control.

When regarding the co-culture immunomodulation model, not only was cytokine production evaluated, but the membrane integrity and metabolic activity of the RAW cells in the basolateral compartment were accessed. For the last two, the results obtained showed that for the duration of the assay, CMC did not cause significant deleterious effects on membrane integrity (Figure 4a) or on the metabolism of the RAW cells present in the basolateral compartment (Figure 4b).

Interestingly, statistically significant (*p* < 0.05) differences were found between the controls and the conditions with CMC, as TEER values with lower variance were observed in the presence of CMC, even when the inflammatory stimulus was added to the system. These results are in line with those previously reported by Marescotti, Lo Sasso [40], which showed that LPS stimuli to the basolateral compartment of a Caco-2/HT29-MTX membrane did not cause any TEER decrease or increase in membrane permeation, with a possible explanation being a dysregulated LPS signaling through the down-regulation of the MD-2 and TLR4 receptors, as previously described by Abreu, Vora [41].

**Figure 5:**
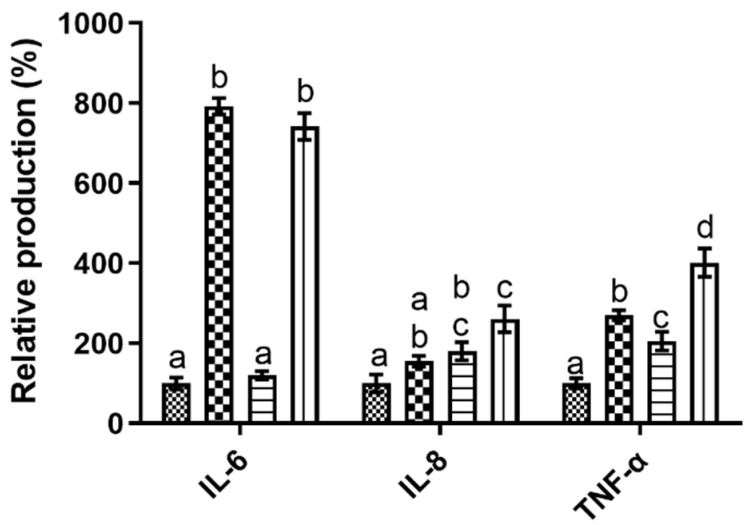
Cytokine production in the RAW complex co-culture model apical side by the Caco-2/HT29-MTX membrane. Different letters represent the statistically significant differences (*p* < 0.05) found between conditions for each cytokine.

**Figure 6:**
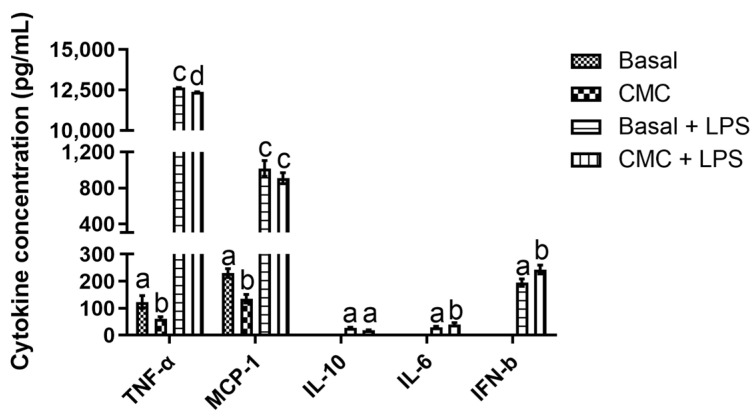
Cytokine production in the RAW complex co-culture model basolateral compartment produced by RAW cells and detected by 13-analyte mouse multiplex panel. Different letters represent the statistically significant differences (*p* < 0.05) found between conditions for each cytokine.

The results obtained regarding the immunomodulatory data can be divided in accordance with their origin: apical data obtained from the Caco-2/HT29-MTX membrane (Figure 5) and basolateral data obtained from the RAW cells (Figure 6). When considering the apical data, it is possible to see in Figure 5 that CMC’s presence led to a general increase in the secretion of pro-inflammatory cytokines with and without LPS stimulus.

Of the analyzed targets, IL-6 presented the highest promotions, with a curious pattern appearing as LPS presence did not lead to statistically significant (*p* > 0.05) differences, contrary to the statistically significant (*p* < 0.05) differences found between conditions with and without CMC. In fact, CMC’s effect on IL-6 production was so strong that, in its presence, this cytokine content was 692% and 642% (without and with LPS) above that of the basal control. This particular result is of concern, as IL-6 elicits an acute phase response and activates humoral and cellular responses via end-stage β-cell differentiation and T-cell activation, leading to the transition from an acute response to chronic inflammation [29]. Furthermore, IL-6-exacerbated secretion in intestinal epithelial cells (IEC) has been associated with intestinal inflammatory processes and shown to play a critical role in IBD and Chron’s disease [29]. When considering IL-8 and TNF-α values on the apical side, two different patterns of response were observed. For the first, it is interesting to note that CMC’s presence did not lead to statistically significant (*p* < 0.05) increases in this cytokine secretion relative to the basal and stimulation control. Furthermore, despite the lack of differences, CMC appeared to have a synergistic effect, with LPS as the highest relative production was registered in this condition. These IL-8 results are quite interesting as, in this particular co-culture model, the presence of increased levels of TNF-α in the basolateral compartment, as here observed, has been linked to the increased secretion of IL-8 in the apical compartment [16,42,43]. However, while this known interaction explains the statistically significant (*p* < 0.05) differences found between the basal control and the inflammation control, they do not explain the increases in both controls observed in the presence of CMC. Once again, this promotion found in the presence of CMC is worthy of record, as IL-8 is an early inflammatory marker due to its initiation of inflammatory cascades and mobilization of neutrophils and T lymphocytes, which will initiate the next steps in intestinal innate immune response, thus playing a key role in IBD [44,45]. Finally, analysis of the TNF-α data obtained showed that, once again, CMC’s presence led to statistically significant (*p* < 0.05) higher levels of this cytokine relative to the controls. For this cytokine, and as seen for IL-8, a synergistic effect could be observed between the inflammatory stimuli and CMC, with the highest relative production of TNF-α being observed in this condition. Considering that this cytokine is a known pro-inflammatory agent linked to the pathogenesis of IBD, chronic gut inflammation and the onset of Crohn’s disease in the intestine [46,47], the data reported here for CMC shows that this compound may pose a problem for gut health. Overall, the data reported for the apical compartment presents a picture that shows CMC as possessing a pro-inflammatory potential in the intestinal lumen, a behavior that has been attributed recently in the literature for CMC but for different reasons. In fact, in vivo works have depicted CMC as being responsible for the removal of the mucous layer that protects the gut mucosa, leading to the building of exacerbated microbial loads in the mucosa and, consequently, small bowel inflammation and IBD development [5,8,48]. If one considers the bibliographic data available and the data obtained here, the picture that emerges is that in addition to what has been published, CMC may also exert a pro-inflammatory effect upon the intestinal cell wall and, thus, it is likely that the reported deleterious effects of CMC are a result of both effects combined.

When considering the effects on the basal compartment (Figure 6), the data obtained showed that of the thirteen targets analyzed, only five were detected in the multiplex assay, with three being pro-inflammatory (TNF-α, MCP-1 and IL-6) and the other two being anti-inflammatory.

For the first group, the standout result was registered for TNF-α, where a strong production was observed in the presence of the inflammatory stimulus, a behavior which has been previously described in the literature [16,42,43]. Interestingly, CMC’s presence in the apical compartment led to statistically significant (*p* < 0.05) lower values of TNF-α both in the presence and absence of LPS. These results are relevant as this cytokine has been widely associated with Crohn’s disease, gut inflammation, IBD and other pathologies, such as obesity, diabetes and colonic cancer. Thus, these reductions associated with CMC’s presence in the intestinal lumen may somewhat counteract the direct deleterious effects observed [46,47,49,50]. For the monocyte chemoattractant protein-1 (MCP-1), the results obtained showed, once again, a strong increase in the quantity secreted in the presence of the inflammatory stimulus. This result was in line with the literature, as LPS has been previously described as being responsible for the activation of the Toll-like receptor 4 (TLR-4) in RAW cells, leading, through the activation of various activation cascades, to the production of various pro-inflammatory cytokines and chemokines, among which is MCP-1 [51,52]. Similar to what was observed for TNF-α, CMC’s presence led to lower levels of this chemokine, with this difference being statistically significant (*p* < 0.05) in the absence of inflammatory stimulation. This is particularly interesting as CMC’s presence led to smaller quantities of MCP-1. Considering that this chemokine is essential in macrophage recruiting and its over-expression has been linked to tumor progression in the intestine, ulcerative colitis, IBD, obesity and diabetes, this under-expression in the presence of CMC may be of interest [53,54,55,56]. For IL-6, the first standout result is the lack of detected cytokines in the non-stimulated conditions, which by itself showed that CMC’s presence in the apical compartment did not have a pro-inflammatory effect in the basolateral side of the Transwell. Secondly, in the stimulated conditions, a significantly (*p* < 0.05) higher IL-6 value was observed in the basolateral compartment when CMC was present in the apical side of the system, showing that a possible synergistic effect between the inflammatory stimulus and some metabolites produced in these conditions may be occurring. When one considers that IL-6 production by macrophages in the gut has been linked to intestinal homeostasis and barrier function, IBD, colon cancer and colitis, this apparent stimuli of IL-6 production in the presence of CMC may be problematic [57,58,59,60].

When considering the data obtained for the two anti-inflammatory cytokines (IL-10 and IFN-β) detected, it is interesting to note the increases registered in the presence of CMC, with this augmented production being statistically significant (*p* < 0.05) for IFN-β. This behavior is even more relevant if one considers that IL-10 is associated with the dampening of intestinal inflammation, IBD and ulcerative colitis risk reduction, as this interferon limits the secretion of pro-inflammatory cytokines, deactivates macrophages and inhibits the secretion of Th1-related cytokines [61,62,63]. For IFN-β, the data obtained present some curiosities, as type I interferons are responsible for the inflammatory response to infections, which then modulate the cell cycle to suppress the infection and upregulate antigen presentation to innate immune cells, leading to the activation of T- and B-cells. Similarly, MCP-1 interferon secretion is stimulated through TLR activation by LPS, but curiously, this will also automatically regulate itself, as IFN-β production has been linked with an upregulation of cytochrome B beta chain expression. This will lead to the production of NADPH oxidase 2-derived H_2_O_2_, causing downregulation of TLR7 and culminating in the inhibition of IFN-β production [64,65,66,67]. While in this work, this downregulation is not on display, and the increased secretion in the presence of CMC in the apical side of the model may be of use in an in vivo setting. In fact, type I interferons play a critical role in the suppression of intestinal inflammation and IBD through the regulation of T regulatory (Treg) cell populations levels, with these cells maintaining intestinal homeostasis under the continuous challenge of microorganisms, their downregulation leading to accelerated colitis [68,69,70,71].

## 4. Conclusions

Overall, the data presented here painted a more in-depth and interesting picture of CMC’s effect upon gut health. With recent works pointing to CMC as a potential pro-inflammatory emulsifier and with the emphasis being on its interaction with microbiota and the mucus layer of the gut epithelia, these results showed that the real picture may be, in fact, more complex. The data obtained in monolayer assays showed that CMC presented a strong pro-inflammatory profile, particularly due to the increase of 1924% detected for f TNF-α. In the co-culture models, CMC’s presence in the apical side, which corresponds to the intestinal lumen, presented similar results as a strong pro-inflammatory response in this compartment was observed, with the promotion of IL-6 and IL-8 secretion but without any impact upon membrane health. Regarding the basolateral compartment, there was no impact on hepatic cells and macrophage metabolism. On the other hand, there was a clear modulation of the macrophages’ inflammatory response with a mixed pattern of response being observed as while there was a strong stimulation of the pro-inflammatory cytokines TNF-α and MCP-1, there was also a modest stimulation of the anti-inflammatory IL-10 and IFN-β cytokines. In conclusion, when considering these and the previously published in vivo data, CMC’s usage as a dietary emulsifier should be reconsidered, as, while it did not exhibit systemic deleterious effects, it presented evidence of a pro-inflammatory effect upon the gut epithelia and lumen, which by itself may play a key role in the potential development of inflammatory gut-related diseases.

## Data Availability

The data presented in this study are available on request from the corresponding author. The data are not publicly available due to confidentiality agreements.
